# Blood-based biomarkers of Alzheimer’s disease and olfactory decline over 15 years in older adults

**DOI:** 10.1007/s11357-025-02038-1

**Published:** 2025-12-17

**Authors:** Ingrid Ekström, Davide Liborio Vetrano, Martina Valletta, Robert Ruane, Maria Larsson, Claudia Fredolini, Bengt Winblad, Giulia Grande, Erika J. Laukka

**Affiliations:** 1 Aging Research Center, Department of Neurobiology, Care Sciences and Society, Karolinska Institutet and Stockholm University, Stockholm, Sweden; 2 Stockholm Gerontology Research Center, Stockholm, Sweden; 3 Gösta Ekman Laboratories, Stockholm University, Stockholm, Sweden; 4 Affinity Proteomics Stockholm, Science for Life Laboratory, Department of Protein Science, School of Engineering Sciences in Chemistry, Biotechnology and Health (CBH), Royal Institute of Technology (KTH), Solna, Sweden; 5 Division of Neurogeriatrics, Department of Neurobiology, Care Sciences and Society, Karolinska Institutet, Solna, Sweden; 6 Theme Inflammation and Aging, Karolinska University Hospital, Huddinge, Sweden

**Keywords:** Alzheimer’s disease, Olfaction, Blood biomarkers, Aging, Longitudinal cohort, Neurodegeneration

## Abstract

Olfactory impairment is common in older age and is a known early feature of several dementia diseases. Blood-based biomarkers of Alzheimer’s disease (AD) now offer a scalable method for detecting pathophysiological mechanisms related to olfactory decline in the general population. However, few studies have examined how these biomarkers relate to long-term olfactory trajectories. Most existing work has been limited to cross-sectional settings. In this population-based study, we used biomarker data collected at baseline and followed participants for up to 15 years, enabling us to test whether early biological changes are temporally linked to subsequent olfactory decline. Data came from the ongoing Swedish National Study on Aging and Care in Kungsholmen (SNAC-K), a longitudinal population-based study with baseline assessments from March 21, 2001, through August 30, 2004. We included participants without prevalent neurodegenerative diseases who completed olfactory assessment at baseline. The 15-year follow-up was finished in December 2019. Data were analysed from December 2023 to April 2024. Serum-derived biomarkers of tau phosphorylated at threonine 217 (p-tau217) and at theorine181 (p-tau181), total tau (t-tau), amyloid-β ratio (Aβ42/Aβ40), neurofilament light chain (NfL), and glial fibrillary acidic protein (GFAP) were obtained at baseline. Linear mixed models examined associations between biomarker quartiles and Sniffin’ Sticks odor identification performance over 15 years, adjusting for demographics, health conditions, and semantic knowledge. We included 1868 participants (mean [SD] age 71.3 [9.9] years; 1122 females [60.1%]). In fully adjusted models, higher quartiles of p-tau217, p-tau181, NfL, and GFAP, and lower quartiles of Aβ42/Aβ40, were associated with steeper olfactory decline, with the steepest decline among participants in the highest quartiles (β for Q4 vs Q1: -0.20 [95% CI: -0.26 to -0.15] for p-tau217; -0.19 [95% CI: -0.25 to -0.13] for p-tau181; -0.23 [95% CI: -0.29 to -0.17] for NfL; β = -0.17 [95% CI: -0.23 to -0.11] for GFAP. Participants in the lowest Aβ42/Aβ40 quartile declined more steeply than those in the highest (β = -0.09 [95% CI: -0.14 to -0.04]). Associations appeared stronger in the oldest participants, in *APOE* ε4 carriers for p-tau181, in non-carriers for NfL and GFAP, and among former smokers for NfL. Blood-based biomarkers of AD were consistently associated with faster olfactory decline in older adults, particularly in the highest biomarker quartiles. These results provide large-scale longitudinal evidence, across up to 15 years of follow-up, that olfactory decline in the general population is linked to AD-related blood biomarkers, supporting the hypothesis that common olfactory losses in ageing partly reflect dementia-related processes.

**Graphical Abstract:**

**Figure:**
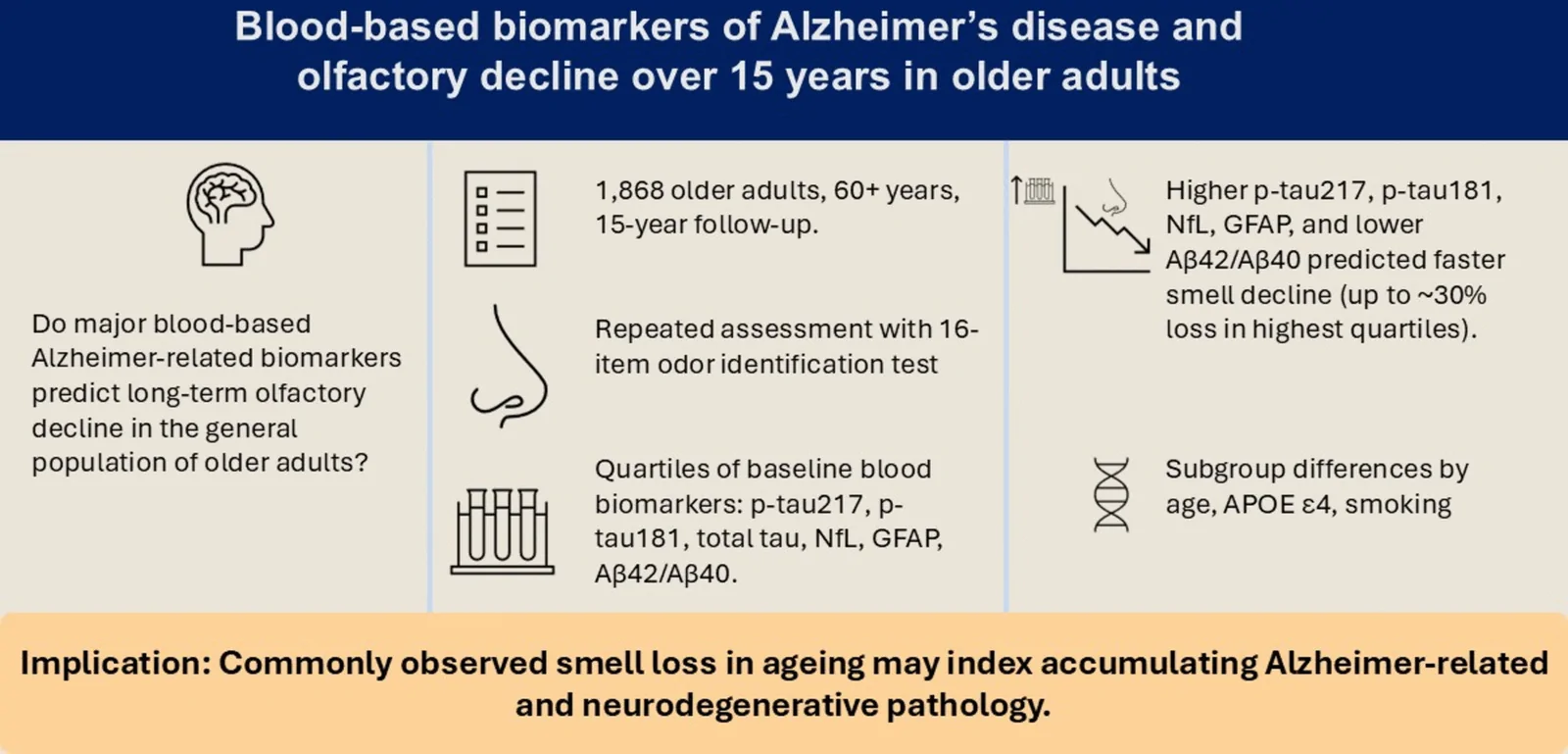

**Supplementary Information:**

The online version contains supplementary material available at https://doi.org/10.1007/s11357-025-02038-1.

## Introduction

Olfactory impairment is common in ageing, affecting approximately 25% of adults aged 60 and above, with higher prevalence with increasing age [1, 2]. Although the precise mechanisms are not yet understood, age-related olfactory loss has been attributed to a range of factors, including genetic susceptibility, peripheral dysfunction, neuroinflammation, environmental exposures, and systemic health conditions [3]. Importantly, it has long been hypothesized that dementia-related pathology may be a major contributor to the olfactory decline commonly observed among older adults [4].

Olfactory impairment is pronounced in several neurodegenerative diseases, including Parkinson’s disease (PD), Alzheimer’s disease (AD), dementia with Lewy Bodies (DLB), and frontotemporal dementia (FTD) [5–7]. Population-based studies have consistently found olfactory deficits to predict the development of all-cause dementia, typically with large effect sizes [8–12] and high accuracy [13]. Obtained associations compare well to sensitivity and specificity of measures obtained by cerebrospinal fluid (CSF) biomarkers when cognitive symptoms are already present [11], positioning olfactory dysfunction as an early and accessible marker of neurodegenerative disease. However, as research and clinical contexts increasingly adopt biomarker-based definitions of dementia diseases [14], there is a critical need to clarify how dementia-related pathophysiological processes relate to olfactory loss in ageing.

So far, the hypothesis that olfactory loss in ageing may partially reflect the accumulation of dementia-related pathology has been difficult to test. Although several studies using CSF or positron emission tomography (PET) have linked olfactory function to biomarkers of mainly tau pathology, these investigations have primarily relied on small or clinical samples and have often been cross-sectional in design [15–21]. Findings have also been mixed, with some studies reporting associations and others finding no clear link. One larger longitudinal PET study found that faster accumulation of tau and amyloid was associated with steeper declines in olfactory ability over a two-year period in community-dwelling adults [22]. With dementia-related pathophysiology beginning to accumulate decades before dementia onset [23], further investigations with longer follow-up periods are warranted. Moreover, it remains unclear how pathophysiological processes relate to factors that have been associated with olfactory impairment in older adults, including, most notably, age, sex, apolipoprotein E (*APOE*) ε4 carrier status or smoking [24–26]. Smoking has been linked to olfactory dysfunction through both reversible peripheral effects and potential long-term contributions to neurodegenerative processes, including increased risk for dementia and neurovascular injury [27–29].

Despite their value, CSF and PET biomarkers pose practical limitations for large-scale or repeated assessments. Recently, blood-based biomarkers have emerged as a promising alternative, enabling the detection of pathological processes, including protein aggregation or axonal injury, in a less invasive and more scalable manner [30, 31]. However, blood-based markers have rarely been examined in relation to olfactory function and most existing studies have relied on cross-sectional designs. For example, a recent large-scale study found that poorer olfactory function was associated with higher plasma levels of phosphorylated tau 181 (p-tau181), glial fibrillary acidic protein (GFAP), and neurofilament light chain (NfL), and lower amyloid-beta 42 to amyloid-beta 40 ratio (Aβ42/Aβ40)(32). Notably, previous studies have not yet examined phosphorylated tau 217 (p-tau217) in relation to olfactory function, which is currently considered one of the most sensitive plasma biomarkers of AD-related pathology in the community [33, 34]. Most importantly, to our knowledge, no study has examined longitudinal olfactory decline in relation to blood-based biomarkers among community-dwelling older adults. Clarifying this temporal relationship is important for understanding whether olfactory decline in ageing, as observed in the general population, partly reflects underlying dementia-related pathology. This knowledge can critically refine how age-related olfactory loss is interpreted in the context of dementia disease.

Using data from 1,868 community-dwelling older adults in the Swedish National Study on Aging and Care in Kungsholmen (SNAC-K), we examined whether baseline levels of serum p-tau217, p-tau181, total tau (t-tau), Aβ42/Aβ40, NfL, and GFAP were associated with trajectories of olfactory identification performance over a 15-year period. We further tested whether sex, age, *APOE* ε4 status, and smoking, modified the associations between biomarkers and olfactory decline.

## Methods

### Participants

For this longitudinal cohort study, participants were recruited within the SNAC-K project, initiated in 2001. Out of 4590 individuals invited to SNAC-K, 3363 enrolled in the baseline assessment conducted between 2001 and 2004. A total of 2848 completed a neuropsychological test battery including the olfactory assessment. We excluded 179 participants with baseline diagnoses of dementia, PD, developmental disorder, or schizophrenia, as well as those with a Mini-Mental State Examination (MMSE) score below 24. Among the remaining 2669 participants, 2475 completed olfactory testing at baseline. Of these, 607 lacked available blood sampling, resulting in a final sample of 1868 participants. Follow-up assessments were conducted every six years between age 60 and 78 years (*n* = 1,270, 66.1%) and every three years for participants aged 78 years and older (*n* = 651, 33.9%). Participants initially under age 78 years were re-assessed every three years as soon as reaching age 78 years. 408 participants completed the olfactory assessment at 3 years, 1,242 at 6 years, 340 at 9 years, 810 at 12 years, and 265 at 15 years. 467 participants did not participate in any follow-up assessment. Participants with available follow-up data were on average younger, had more years of education, and higher scores on the MMSE and the odor identification test at baseline (*p*s < 0.001). No significant difference regarding sex was found between the samples (*p* = 0.114).

### Data collection

At each visit, standardized protocols were followed for data collection, including face-to-face interviews, clinical, laboratory, and neuropsychological examinations administered by trained professionals. Evaluations took place at the research center, participants' homes, or institutions for those unable to travel.

### Assessment of covariates and modifying factors

Demographic data was obtained during an interview by a nurse. Age was measured in years since birth, and education in years of schooling. As odor identification may partly depend on semantic memory ability [35] we controlled for word knowledge measured by the SRB1 Swedish vocabulary test. Venous blood samples were taken for DNA extraction and *APOE* allele genotyping, categorizing participants as ε4 carriers if they had one or two alleles or non-carriers if they had none.

Participants' health status assessments, detailed elsewhere [36], involved medical history collection, physician interview, general and neurological examinations, diagnostic tests, and data from medical records and the Swedish National Patient Register. Diagnoses were coded using ICD-10. Diseases related to olfactory function [37] and blood biomarkers [38] in SNAC-K were assessed as potential covariates, including cerebrovascular disease, cardiovascular disease burden (number of conditions including atrial fibrillation, heart failure, and ischaemic heart disease; range 0 to 3), chronic kidney disease, obesity, and anaemia. Smoking status was based on self-reported information and categorized as never, former, or current smoker.

The clinical diagnosis of dementia followed DSM-IV criteria [39]. Diagnoses were based on comprehensive assessments at each test-wave, including medical and drug history, general and neurological examinations (including Parkinsonism assessment), cognitive testing (MMSE, clock drawing, Digit Span, and items addressing e.g. problem-solving, abstract thinking, and orientation), and evaluations of daily functioning. Diagnoses were made in a three-step process: an initial assessment by the examining physician, an independent review by a second physician blinded to the first diagnosis, and adjudication by senior neurologists in cases of disagreement. For participants who died between follow-ups, clinical records and death certificates were reviewed by the same diagnostic team.

### Blood biomarker assessment

The procedure to obtain the biomarker values from blood serum is detailed elsewhere [39]. In brief, peripheral venous blood samples were collected at baseline and stored at − 80 °C until analysis. The analyses were conducted at the Affinity Proteomics Stockholm Unit (SciLifeLab), using the Quanterix SR-X (software version 1.2.0) Biomarker Detection System. The Simoa Neuro 3-plex A Kit (Quanterix, cat. no. 101995) was used to measure serum Aβ40, Aβ42, and t-tau levels, and the Simoa p-tau181 Advantage V2 Kit (Quanterix, cat. no. 103714)) was used to measure serum p-tau181 levels. The commercial assay Simoa ALZpath p-tau217 Advantage PLUS (Quanterix, cat. no. 104570), developed for the Quanterix HD-X system, was used to measure p-tau217. Serum concentrations of NfL and GFAP were measured using the Simoa Neuro 2-plex B Kit (Quanterix, cat. no. 103520).

### Odor identification assessment

Odor identification was assessed with the Sniffin’ Sticks, a well-established norm-referenced test with high test–retest reliability [40, 41]. The testing procedure has been described in detail elsewhere [25, 35]. Briefly, participants are presented with 16 felt tip-pens containing common household odors. They are asked to freely identify each odor by providing a verbal descriptor (free identification). If they fail to give a correct label, they are instructed to pick the option that best matches the odor out of four written alternatives (cued identification). In case of missing information for a specific item, a score of 0.25 was given (equivalent to chance-level performance). The odor identification score represents the number of correctly identified odors with either free or cued identification (range 0–16). Testing order of the odor items was randomized into three different test versions. Participants with self-reported anosmia or obvious symptoms of viral infections or respiratory problems were excluded from testing.

### Statistical analysis

We employed linear mixed-effects models with unstructured variance–covariance matrices to examine associations between blood biomarkers and olfactory change. Time in study (years) served as the time scale, with random intercept and slope accounting for individual differences over time. Missing olfactory values during follow-up were imputed using maximum likelihood. The main analysis evaluated quartiles of each biomarker in relation to olfactory ability and decline. For Aβ42/Aβ40, the highest quartile was used as reference, whereas for the other biomarkers the lowest quartile was reference. Quartiles were used to facilitate interpretation and visualization of dose–response relationships. As a secondary analysis, biomarkers were modeled as continuous variables to assess whether results were consistent across specifications. To evaluate the magnitude of associations, model-based predicted odor identification scores were derived from the fully adjusted mixed-effects models at baseline and after 15 years of follow-up. These estimates represent expected trajectories under the fitted models. Differences in decline between the highest and lowest biomarker quartiles (Q4 vs Q1) were quantified using linear combinations of model coefficients, with 95% confidence intervals calculated via the delta method.

All models were first adjusted for age, sex, and education, and then additionally for vocabulary, cardiovascular disease, chronic kidney disease, cerebrovascular disease, and anemia, based on their associations with olfactory decline at p < 0.10. Continuous covariates were mean-centered.

Sensitivity analyses included (i) repeating models after excluding participants without olfactory follow-up data, and (ii) excluding participants who developed dementia during follow-up. The latter step aimed to evaluate whether biomarker-olfaction associations were driven by incipient dementia.

Potential effect modification was examined by stratifying models according to age group (< 78 vs ≥ 78 years), sex, smoking status, and *APOE* ε4 carrier status. For the effect modification analyses, we used continuous biomarker values rather than quartiles. This approach retains statistical power, avoids arbitrary cut-points, and enables testing whether the strength of the biomarker–olfaction association varies across subgroups (age, sex, *APOE* ε4 status, smoking). We formally tested interactions by including cross-product terms between biomarker levels and the subgroup variable.

Analyses were conducted using STATA version 17. No adjustments were made for multiple comparisons [42]. Reported results include 95% confidence intervals where applicable.

## Results

### Sample characteristics

We included 1,868 participants (mean [SD] age, 71.3 [9.9] years; 1122 females [60.1%] and 746 males [39.9%]). Background characteristics for the total sample and by age group are summarized in Table 1 and by follow-up status in Supplementary Table 1. Mean follow-up time was 7.5 [2.3] years for the total sample. Estimated average performance in odor identification over follow-up decreased significantly as a function of time in the whole sample (annual change rate of −0.21; 95% CI: −0.23 to −0.19).

**Table 1:** Baseline characteristics of the total sample and by age group

Characteristic	Total (*n* = 1868)	Age < 78 years (*n* = 1241)	Age ≥ 78 years (*n* = 627)
Odor identification, *M* (SD)	11.81 (2.93)	12.66 (2.35)	10.15 (3.25)
Range	1 to 16	2 to 16	1 to 16
Age, *M* (SD)	71.29 (9.86)	65.24 (4.84)	83.28 (5.16)
Range	58.44 to 100.9	58.44 to 74.4	78.12 to 100.9
Education (years), *M* (SD)	12.39 (4.24)	13.26 (4.15)	10.66 (3.86)
Range	3 to 31.5	3 to 31.5	3 to 26
MMSE, *M* (SD)	28.95 (1.28)	29.28 (0.96)	28.29 (1.54)
Range	24 to 30	24 to 30	24 to 30
SRB, *M* (SD)	23.06 (4.90)	24.00 (4.35)	21.17 (5.37)
Range	1 to 30	1 to 30	3 to 30
Sex, no. (%)—men	746 (39.9%)	541 (43.6%)	205 (32.7%)
Sex, no. (%)—women	1122 (60.1%)	700 (56.4%)	422 (67.3%)
Smoking, current (%)	289 (15.6%)	235 (19.1%)	54 (8.6%)
Smoking, ever (%)	746 (40.2%)	531 (43.1%)	215 (34.4%)
Smoking, never (%)	823 (44.3%)	466 (37.8%)	357 (57.0%)
*APOE* ε4 status, no. (%)—no E4	1279 (70.2%)	829 (68.7%)	450 (73.3%)
*APOE* ε4 status, no. (%)—E4	542 (29.8%)	378 (31.3%)	164 (26.7%)
Kidney disease, no. (%)—absent	1323 (70.8%)	1072 (86.4%)	251 (40.0%)
Kidney disease, no. (%)—present	545 (29.2%)	169 (13.6%)	376 (60.0%)
Cerebrovascular disease, no. (%)—absent	1777 (95.1%)	1203 (96.9%)	574 (91.6%)
Cerebrovascular disease, no. (%)—present	91 (4.9%)	38 (3.1%)	53 (8.4%)
Anaemia, No. (%)—absent	1703 (91.2%)	1191 (96.0%)	512 (81.7%)
Anaemia, No. (%)—present	165 (8.8%)	50 (4.0%)	115 (18.3%)
Obesity, No. (%)—absent	1610 (86.2%)	1043 (84.0%)	567 (90.4%)
Obesity, No. (%)—present	258 (13.8%)	198 (16.0%)	60 (9.6%)
Cardiovascular disease, no. (%)—score 0	1508 (80.7%)	1105 (89.0%)	403 (64.3%)
Cardiovascular disease, no. (%)—score 1	257 (13.8%)	115 (9.3%)	142 (22.7%)
Cardiovascular disease, no. (%)—score 2	83 (4.4%)	20 (1.6%)	63 (10.1%)
Cardiovascular disease, no. (%)—score 3	20 (1.1%)	1 (0.1%)	19 (3.0%)

*Note.* Values are presented as mean (SD) or number (%). APOE ε4 status missing for 47 participants; smoking status missing for ten participants

### Quartiles of blood biomarkers and olfactory change

At baseline, most biomarkers were not consistently associated with odor identification scores, either in basic models or after full covariate adjustment. A modest cross-sectional association was observed only for p-tau217, where individuals in the highest quartile had lower odor scores compared with those in the lowest quartile (Table 2).

**Table 2:** Results of basic and fully adjusted linear models estimating odor identification scores as a function of biomarker quartiles for the total sample

		Basic adjusted model^a^	Fully adjusted model^b^	Basic adjusted model^a^	Fully adjusted model^b^
		Baseline	Baseline	Predictor × time	Predictor × time
		*β* (95% CI)	*β* (95% CI)	*β* (95% CI)	*β* (95% CI)
**Aβ-ratio**	Q3	0.098 (− 0.218, 0.414)	0.102 (− 0.211, 0.415)	− 0.002 (− 0.048, 0.045)	− 0.001 (− 0.048, 0.045)
	Q2	0.024 (− 0.303, 0.352)	0.050 (− 0.274, 0.374)	− 0.082 (− 0.131, − 0.033)**	− 0.080 (− 0.130, − 0.031)**
	Q1	− 0.081 (− 0.413, 0.251)	− 0.010 (− 0.339, 0.319)	− 0.087 (− 0.137, − 0.0357)**	− 0.088 (− 0.139, − 0.037)**
*P* trend		n.s	n.s	< 0.001	< 0.001
**P-tau217**	Q2	− 0.233 (− 0.559,.093)	−.0221 (− 0.545, 0.102)	− 0.040 (− 0.086, 0.006)	− 0.039 (− 0.085, 0.008)
	Q3	− 0.097 (− 0.429, 0.236)	− 0.160 (− 0.490, 0.170)	− 0.100 (− 0.146, − 0.054)***	− 0.099 (− 0.145, − 0.054)***
	Q4	− 0.045 (− 0.823, − 0.083)*	− 0.414 (− 0.785, −.0433)*	− 0.120 (− 0.255, − 0.144)***	− 0.202 (− 0.258, −.146)***
*P* trend		0.046	< 0.001	n.s	< 0.001
**P-tau181**	Q2	− 0.094 (− 0.408, 0.220)	− 0.067 (− 0.379, 0.244)	− 0.028 (− 0.073, 0.016)	− 0.027 (− 0.072, 0.018)
	Q3	− 0.182 (− 0.510, 0.146)	− 0.218 (− 0.543, 0.107)	− 0.082 (− 0.129, −.036)**	− 0.081 (− 0.128, − 0.035)**
	Q4	− 0.222 (− 0.596, 0.152)	− 0.143 (− 0.517, 0.230)	− 0.184 (− 0.243, − 0.125)***	− 0.187 (− 0.246, − 0.128)***
*P* trend		n.s	n.s	< 0.001	< 0.001
**T-tau**	Q2	0.125 (− 0.192, 0.442)	0.056 (− 0.258, 0.371)	− 0.007 (− 0.055, 0.040)	− 0.007 (− 0.055, 0.041)
	Q3	− 0.041 (− 0.361, 0.279)	− 0.048 (− 0.365, 0.269)	− 0.040 (− 0.087, 0.011)	− 0.039 (− 0.088, 0.010)
	Q4	− 0.244 (− 0.577, 0.089)	− 0.266 (− 0.598, 0.067)	0.014 (− 0.038, 0.066)	0.012 (− 0.040, 0.065)
*P* trend		n.s	n.s	n.s	n.s
**NfL**	Q2	− 0.025 (− 0.337, 0.287)	− 0.023 (− 0.331, 0.286)	− 0.087 (− 0.128, − 0.045)***	− 0.087 (− 0.129, − 0.045)***
	Q3	− 0.059 (− 0.419, 0.301)	− 0.109 (− 0.468, 0.249)	− 0.141 (− 0.189, − 0.093)***	− 0.138 (− 0.186, − 0.090)***
	Q4	− 0.350 (− 0.799, 0.098)	− 0.334 (− 0.793, 0.124)	− 0.230 (− 0.296, − 0.164)***	− 0.234 (− 0.300, − 0.169)***
*P* trend		n.s	n.s	< 0.001	< 0.001
**GFAP**	Q2	− 0.035 (− 0.349, 0.280)	− 0.074 (− 0.385, 0.237)	− 0.015 (− 0.059, 0.029)	− 0.013 (− 0.057, 0.032)
	Q3	0.026 (− 0.314, 0.367)	− 0.019 (− 0.356, 0.319)	− 0.072 (− 0.119, − 0.024)*	− 0.072 (− 0.120, − 0.024)
	Q4	− 0.176 (− 0.571, 0.219)	− 0.226 (− 0.620, 0.167)	− 0.171 (− 0.231, − 0.111)***	− 0.174 (− 0.235, − 0.114)
*P* trend		n.s	n.s	< 0.001	< 0.001

Note. For the Aβ42/40 ratio, quartiles are presented in reverse order relative to other biomarkers (Q1 = lowest ratio/highest amyloid burden; Q3 = highest ratio/lowest amyloid burden)

*CI* confidence interval

^***^p < 0.001, **p < 0.01, *p < 0.05

^a^Adjusted for age, sex, and years of education. *N* = 1867

^b^Adjusted for age, sex, years of education, vocabulary (SRB:1), chronic kidney disease, anaemia, cerebrovascular disease, and cardiovascular disease. *N* = 1856

**Fig. 1:**
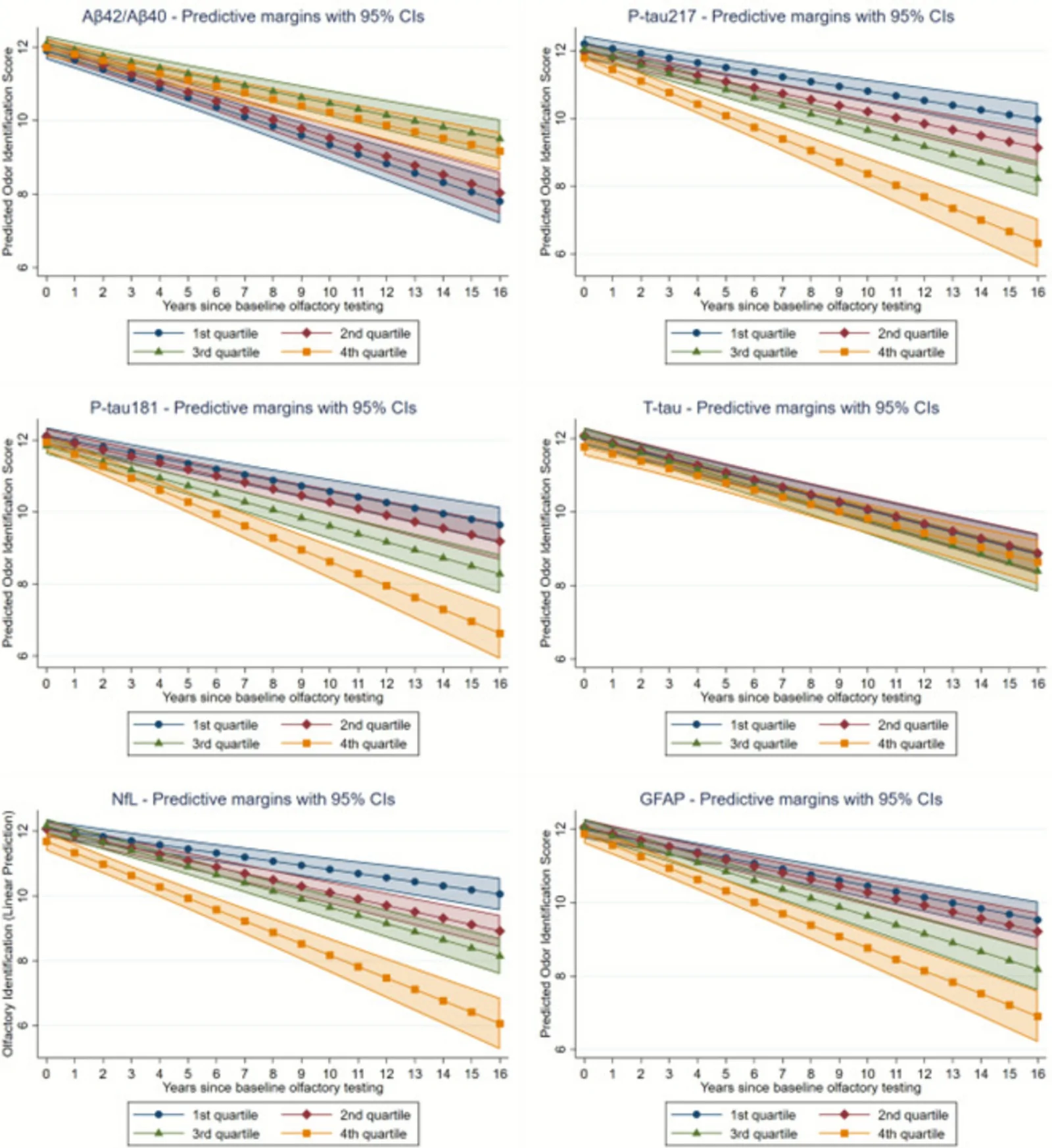
Predicted trajectories of odor identification performance by biomarker quartiles over 15 years of follow-up. Results are based on fully adjusted linear mixed-effects models for Aβ42/Aβ40, p-tau217, p-tau181, total tau, NfL, and GFAP. Higher quartiles reflect higher biomarker concentrations, except for Aβ42/Aβ40 where higher quartiles reflect lower pathology (i.e., higher Aβ42/Aβ40 ratio). The *y*-axis represents predicted odor identification scores, and the *x*-axis represents years since baseline olfactory assessment. Shaded areas indicate 95% confidence intervals

In basic models adjusted for age, sex, and education, higher quartiles of p-tau217, p-tau181, NfL, and GFAP, as well as lower quartiles of Aβ42/Aβ40, were each associated with steeper decline in odor identification. These associations persisted after additional adjustment for vocabulary, cardiovascular disease, cerebrovascular disease, chronic kidney disease, and anemia. Total tau was not consistently associated with olfactory decline in either model. Marked dose–response gradients were observed for p-tau217, p-tau181, and NfL. Participants in the highest quartiles of these biomarkers showed the steepest trajectories of olfactory loss across follow-up (Table 2). No consistent associations were observed for total tau. Results are summarized in Table 2 and illustrated in Fig. 1.

For most biomarkers, model-based estimates indicated that participants with high biomarker levels were predicted to lose about twice to three times as many odor items as those with low biomarker levels. For p-tau217, participants in the lowest quartile were estimated to decline by approximately 2.1 items over 15 years (from 12.2 to 10.1), compared with 5.1 items in the highest quartile (from 11.8 to 6.7), corresponding to a Q4-Q1 difference of 3.0 items (95% CI: 2.2–3.9). For p-tau181, the decline was 2.3 items in Q1 (from 12.1 to 9.8) and 5.1 items in Q4 (from 12.0 to 6.8), with a difference of 2.8 items (95% CI: 1.9–3.7). For NfL, participants in Q1 declined by 1.9 items (from 12.1 to 10.2), whereas those in Q4 declined by 5.4 items (from 11.8 to 6.3), corresponding to a difference of 3.5 items (95% CI: 2.5–4.5).

For GFAP estimated decline was 2.5 items in Q1 (from 12.1 to 9.6) and 5.1 items in Q4 (from 11.8 to 6.7), yielding a difference of 2.6 items (95% CI: 1.7–3.5). For Aβ42/Aβ40, the pattern was inverse: participants in the lowest quartile (greater amyloid pathology) declined by 3.9 items (from 11.9 to 8.1), compared with 2.6 items in the highest quartile (from 12.0 to 9.4), corresponding to a difference of 1.3 items (95% CI: 0.6–2.1).

### Sensitivity analyses

After excluding 467 participants without follow-up olfactory assessments, associations with rate of decline were essentially unchanged (Supplementary Table 2). Higher quartiles of p-tau181, p-tau217, NfL, and GFAP remained associated with faster decline, and lower quartiles of Aβ42/Aβ40 continued to predict steeper decline.

During follow-up, 312 participants (16.7%) developed dementia. Supplementary Table 1 presents baseline characteristics for participants who developed dementia during follow-up and those who remained dementia-free. Group comparisons with the total sample showed that individuals who later developed dementia were older at baseline and had lower educational attainment, poorer olfactory identification performance, lower MMSE scores, and lower vocabulary scores than the total baseline sample (all *p* < 0.001). They were also more likely to be *APOE* ε4 carriers and to have chronic kidney disease, cerebrovascular disease, anaemia, and a higher cardiovascular disease burden (all *p* < 0.01). Women were overrepresented among those who developed dementia (*p* = 0.009).

After excluding these individuals, the overall pattern was preserved but effect sizes were attenuated (Supplementary Table 2). For Aβ42/Aβ40, the associations were considerably weaker, with only partial evidence of faster decline in the lower quartiles. For p-tau217, higher quartiles continued to predict steeper decline, but the effect sizes were smaller than in the total sample. For p-tau181, the association was weaker and largely confined to the highest quartile. Associations for NfL and GFAP were also reduced in magnitude compared with the full sample, although higher quartiles still predicted faster decline.

### Modifying factors

When biomarkers were examined as continuous variables, findings were consistent with the quartile analyses, with higher p-tau181, p-tau217, NfL, and GFAP, and lower Aβ42/Aβ40 associated with faster olfactory decline, while no association was observed for total tau (Supplementary Table 3). Formal tests of interaction showed significant interactions with age group for GFAP (*p* = 0.047) and p-tau181 (*p* = 0.032), both indicating stronger associations with olfactory decline among participants aged 78 years and older. For sex, none of the interaction terms reached statistical significance. For *APOE* ε4 carrier status, interaction tests showed significant interactions for NfL, GFAP, and p-tau181. The association between p-tau181 and olfactory decline was stronger in *APOE* ε4 carriers (*p* = 0.038), while associations for NfL and GFAP were stronger in non-carriers (*p*s < 0.05). For smoking status, the interaction between NfL and former smoking reached statistical significance (*p* = 0.01), indicating a stronger association between NfL and olfactory decline among former smokers.

Stratified analyses according to age group, *APOE* ε4 carrier status, and smoking status are summarized in Table 3.

**Table 3:** Results of fully adjusted linear model^a^ estimating odor identification scores as a function of continuous biomarker levels in sub-samples

	Predictor × time					
Subgroup	Aβ42/Aβ40	P-tau217	P-tau181	T-tau	NfL	GFAP
	*β* (95% CI)	*β* (95% CI)	*β* (95% CI)	*β* (95% CI)	*β* (95% CI)	*β* (95% CI)
**Age < 78 (*n* = 1241)**	0.024 (− 0.014, 0.061)	− 0.145*** (− 0.230, − 0.060)	− 0.090** (− 0.155, − 0.025)	0.012 (− 0.011, 0.035)	− 0.065* (− 0.127, − 0.003)	− 0.064* (− 0.128, − 0.001)
**Age ≥ 78 (*n* = 627)**	0.025 (− 0.016, 0.067)	− 0.213*** (− 0.292, − 0.135)	− 0.141*** (− 0.221, − 0.061)	− 0.010 (− 0.073, 0.052)	− 0.086** (− 0.154, − 0.018)	− 0.067* (− 0.133, − 0.001)
**Men (*n* = 746)**	0.033 (− 0.009, 0.075)	− 0.181*** (− 0.267, − 0.095)	− 0.103** (− 0.178, − 0.028)	0.010 (− 0.019, 0.039)	− 0.085** (− 0.156, − 0.014)	− 0.070* (− 0.138, − 0.002)
**Women (*n* = 1122)**	0.022 (− 0.019, 0.063)	− 0.169*** (− 0.247, − 0.092)	− 0.093** (− 0.161, − 0.025)	− 0.010 (− 0.043, 0.022)	− 0.057* (− 0.120, − 0.005)	− 0.050* (− 0.118, − 0.017)
**Never-smokers (*n* = 823)**	0.013 (− 0.016, 0.042)	− 0.187*** (− 0.247, − 0.127)	− 0.091*** (− 0.139, − 0.044)	0.011 (− 0.027, 0.048)	− 0.046* (− 0.090, − 0.003)	− 0.045 (− 0.093, 0.004)
**Former smokers (*n* = 746)**	0.031* (0.003, 0.060)	− 0.180*** (− 0.247, − 0.111)	− 0.070** (− 0.116, − 0.023)	− 0.012 (− 0.050, 0.026)	− 0.146*** (− 0.203, − 0.089)	− 0.071** (− 0.125, − 0.018)
**Current smokers (*n* = 289)**	0.064 (− 0.004, 0.131)	− 0.098 (− 0.202, 0.004)	− 0.147* (− 0.278, − 0.017)	0.008 (− 0.033, 0.049)	− 0.015 (− 0.059, 0.029)	− 0.002 (− 0.031, 0.026)
**No *APOE* ε4 (*n* = 1279)**	0.019 (− 0.003, 0.041)	− 0.155*** (− 0.201, − 0.109)	− 0.063** (− 0.098, − 0.027)	0.004 (− 0.024, 0.032)	− 0.078*** (− 0.115, − 0.040)	− 0.054** (− 0.091, − 0.017)
***APOE* ε4 carriers (*n* = 542)**	0.035 (− 0.011, 0.081)	− 0.160*** (− 0.244, − 0.076)	− 0.145*** (− 0.219, − 0.070)	− 0.002 (− 0.037, 0.033)	− 0.027 (− 0.066, 0.011)	− 0.004 (− 0.031, 0.022)

Note. *CI* confidence interval

^***^p < 0.001; ** p < 0.01; * *p* < 0.05

^a^Adjusted for age, sex, years of education, vocabulary (SRB:1), chronic kidney disease, anaemia, cerebrovascular disease, and cardiovascular disease

## Discussion

In this population-based study of 1,868 older adults followed for up to 15 years, we found that lower quartiles of serum Aβ42/Aβ40 and higher quartiles of p-tau217, p-tau181, NfL, and GFAP were associated with steeper olfactory decline, whereas no consistent associations were observed for total tau. These associations persisted after full covariate adjustment and were generally consistent in sensitivity analyses excluding participants without follow-up. When participants who developed dementia were excluded, the overall pattern remained but associations for p-tau217 and especially p-tau181 were attenuated, suggesting that these links may be partly driven by incipient dementia. When biomarkers were analyzed as continuous variables, findings were consistent with the quartile-based results. Effect modification analyses further showed that associations of p-tau181 and GFAP with olfactory decline were stronger among participants aged 78 years and older, that the association between p-tau181 and olfactory decline was more pronounced in *APOE* ε4 carriers, and that associations of NfL and GFAP were stronger among non-carriers. For NfL, a stronger association was also observed among former smokers.

We found that five of the six biomarkers were associated with olfactory decline. The same markers were recently validated in an overlapping cohort, where elevated levels of p-tau181, p-tau217, NfL, and GFAP were associated with increased risk of all-cause and AD dementia during up to 16 years of follow-up [39]. The observed lack of associations between olfactory decline and t-tau is consistent with previous work showing that plasma t-tau demonstrates only weak and inconsistent correlations with AD pathology and clinical phenotypes, suggesting limited utility as an early biomarker compared with phosphorylated tau species [39, 43].

We observed substantial differences in estimated olfactory decline trajectories across quartiles, especially regarding p-tau217, p-tau181, and NfL. In model-based estimates, older adults with the highest concentrations in these biomarkers were predicted to lose approximately 5 to 6 odor items over 15 years, compared with only 1 to 2 items among those within the lowest quartile of biomarker levels, suggesting that olfactory decline may be two- to threefold steeper in the presence of AD-related pathology. Prior studies have identified p-tau181 and p-tau217 as sensitive markers of AD pathology, capturing both tau accumulation and amyloid deposition [44–46]. NfL is a widely established marker of neuroaxonal injury and neurodegeneration [47, 48]. In line with a recent study highlighting p-tau217 and NfL as key markers in AD and progranulin-related neurodegeneration, respectively [49], we found that both biomarkers were similarly predictive of steeper olfactory decline, supporting olfaction as an early marker of neurodegenerative processes across etiologies. Beyond the established AD and neurodegeneration markers, the study also identified several important additional candidate proteins linked to synaptic function, inflammation, and proteinopathy-specific processes [49]. Future work integrating broader proteomic discovery approaches may help identify complementary molecular pathways underlying olfactory decline.Our findings significantly extend previous cross-sectional studies linking dementia-related CSF, PET, and blood markers to olfactory function by demonstrating longitudinal associations over more than a decade. By establishing that AD biomarker elevations precede and predict steeper olfactory decline in the general older population, these results highlight olfactory testing as a sensitive clinical marker of ongoing neurodegenerative processes and provide new evidence for integrating sensory outcomes in biomarker-based dementia research.

It is noteworthy that biomarker associations were consistently observed for longitudinal olfactory decline but were largely absent for baseline performance. In this cognitively healthy sample at study entry, none of the biomarkers showed robust cross-sectional associations, except for p-tau217, where individuals in the highest quartile already exhibited lower olfactory scores. This cross-sectional association for p-tau217 may reflect that tau pathology involves olfactory regions early in the disease process, and it is also consistent with evidence that p-tau217 is a particularly sensitive marker of AD-related pathology [50]. By contrast, baseline olfactory ability is likely influenced by factors unrelated to neurodegeneration, including sinonasal conditions, environmental exposures, and other health-related or experiential factors, which may obscure biomarker-olfaction associations at a single time point. Longitudinal analyses, by capturing within-person change, reduce the impact of such stable influences and provide thus a more sensitive indicator of emerging neurodegenerative processes.

The sensitivity analyses provide important context for interpreting the main findings. Excluding participants without follow-up confirmed that the observed associations were not explained by differential attrition, supporting their robustness. When individuals who later developed dementia were excluded, associations for all biomarkers were reduced in magnitude, confirming that part of the link between biomarker levels and olfactory decline is driven by processes occurring in the preclinical phase of dementia. The attenuation was most pronounced for p-tau181 and Aβ42/Aβ40, consistent with their established role as early markers of ongoing Alzheimer’s pathology [39]. Associations for p-tau217, NfL, and GFAP also weakened but remained evident, suggesting that in addition to ongoing pathology, these markers may reflect mechanisms of olfactory loss that either operate very early in the disease trajectory, long before clinical conversion, or extend beyond prodromal dementia into broader neurodegenerative or systemic ageing-related processes.

Investigation of potential effect modifiers, including age, sex, smoking, and *APOE* carrier status, showed that associations between the biomarkers and olfactory decline were largely consistent across subgroups, with evidence of modification for certain biomarkers. The association between p-tau181 and olfactory decline was strongest among *APOE* ε4 carriers and participants aged 78 years or older. This aligns with prior studies suggesting that olfactory decline interacts with the *APOE* ε4 allele in cognitive decline [51–53], potentially via ε4-related disruptions of sensory-cognitive network integration [54]. The interaction between p-tau181 and *APOE* ε4 corresponds well to regional patterns of AD pathology, as amyloid and tau pathologies originate in entorhinal and anteromedial temporal regions [55, 56], areas essential for olfactory processing [4]. Plasma p-tau181 levels correlate with neurofibrillary tangle pathology, particularly in temporoparietal regions characteristic of advanced Braak stages [57]. By contrast, we did not observe a significant interaction between p-tau217 and *APOE* ε4, despite p-tau217’s high specificity for AD pathology and its strong association with cognitive decline in previous studies [34, 58]. P-tau217 may reflect more widespread neocortical tau pathology [59], while p-tau181 may be more sensitive to medial temporal tau accumulation [60] which interacts with *APOE* ε4 in early disease stages [61].

By contrast, we found that the association between NfL and olfactory decline was strongest among *APOE* ε4 non-carriers and among former smokers. While many prior studies suggested that only current smoking is associated with olfactory dysfunction [25, 29], our findings point to a more durable vulnerability associated with smoking history. Former smoking amplified the association between NfL and olfactory decline, suggesting that smoking-related neurotoxic damage may leave long-lasting effects on the olfactory system. This interpretation is supported by research showing elevated plasma NfL levels even in former smokers [62], suggesting persistent neuroaxonal damage after smoking cessation. The absence of a similar interaction among current smokers was surprising but could reflect survival bias, health-related smoking cessation, or limited statistical power due to a small number of current smokers.

The association between GFAP and olfactory decline was most pronounced among individuals aged 78 years or older and among *APOE* ε4 non-carriers. Although fewer studies have addressed this association, GFAP may reflect astrocyte activation and neuroinflammation [63–65]. Some evidence from PD suggests that astrogliosis may impair olfactory and cognitive function [66]. The stronger associations observed for GFAP and NfL in *APOE* non-carriers may suggest that these markers partly capture neurodegenerative or glial processes that are less directly linked to *APOE*-related AD pathology. However, these patterns should be interpreted with caution, as differences in subgroup composition may also contribute to the observed interactions. It is also important to note in this context that the role of the blood-based biomarker GFAP has recently been questioned, as some studies have raised concerns about its specificity for reactive astrocytes in neurodegenerative diseases [67], making the interpretation of its association with olfactory decline more uncertain.

We did not observe any modifying effects of sex, despite well-documented sex differences in both olfactory function [68] and dementia incidence [69]. It is important to note, however, that sex differences in olfaction have primarily been linked to life-long differences in olfactory ability, whereas evidence for sex differences in olfactory decline during ageing remains limited [25].

This study has major strengths, including the long follow-up, large sample size, and comprehensive assessment of multiple AD and neurodegeneration biomarkers. Attrition was addressed by repeating analyses restricted to participants with olfactory follow-up data, confirming the longitudinal patterns. Nonetheless, generalisability may be affected by the relatively healthy nature of the SNAC-K cohort, potentially leading to conservative estimates. Future studies should aim to replicate these findings in more socioeconomically diverse populations to strengthen external validity. Another key limitation is the lack of available biomarkers for Lewy body pathology. Given the well-established role of Lewy body disease in olfactory dysfunction [70], and the absence of reliable blood-based assays for α-synuclein, this likely represents the strongest limitation of the present study. Our findings provide an important step beyond previous cross-sectional studies by demonstrating that biomarkers of neurodegeneration and AD-related processes are linked to subsequent long-term olfactory decline.

Future studies should examine how longitudinal biomarker trajectories relate to olfactory change, and whether integrating olfactory assessments with blood biomarkers improves early identification of cognitive decline and dementia. Further research on the interaction between AD biomarkers, systemic inflammation, and Lewy body pathology will be critical to disentangle the mechanisms linking olfactory decline and neurodegeneration [37, 71]. Although smoking was incorporated due to its known association with olfactory function, other lifestyle factors relevant for dementia risk (e.g., physical activity, diet, and social engagement [72] were not included because of missingness constraints but should be relevant to explore in future studies. In addition, assessing relationships with biomarkers across olfactory domains such as threshold and discrimination will be important in cohorts with broader sensory testing. Finally, integrating repeated biomarker assessments with regional neuroimaging, particularly in olfactory-relevant structures [32], would help elucidate temporal dynamics and biological pathways linking olfactory decline to neurodegenerative processes.

In summary, in a large population-based cohort of older adults followed for up to 15 years, olfactory decline was robustly associated with blood-based biomarkers of AD and neurodegeneration. The strongest associations were observed for p-tau217, p-tau181, and NfL. Collectively, our findings offer longitudinal evidence that olfactory decline in older adults is linked to AD- and neurodegeneration-related pathology, with variation partially shaped by age, *APOE* genotype, and smoking history.

## Supplementary Information

Below is the link to the electronic supplementary material.

Supplementary file1 (DOCX 40 kb)

Supplementary file2 (DOCX 28 kb)

## Declarations

### Use of AI tools

During the preparation of this work, the authors occasionally used ChatGPT (OpenAI) to shorten the length of specific sentences. After using this tool/service, the authors reviewed and edited the content as needed and take full responsibility for the content of the publication.
