# Supplementary material for: Blood-based biomarkers of Alzheimer’s disease and olfactory decline over 15 years in older adults
Source: GeroScience. 2025 Dec 17;48(5):7245–60. doi: 10.1007/s11357-025-02038-1 (PMC13601474; doi:10.1007/s11357-025-02038-1)
Supplement: Supplementary file 1 [file 11357_2025_2038_MOESM1_ESM.docx]

STROBE Statement

|  | Item No. | Recommendation | Page  No. | Relevant text from manuscript |
| --- | --- | --- | --- | --- |
| **Title and abstract** | 1 | (*a*) Indicate the study’s design with a commonly used term in the title or the abstract | 3 | The abstract includes "longitudinal population-based study" to indicate design. |
|  |  | (*b*) Provide in the abstract an informative and balanced summary of what was done and what was found | 3 | Abstract summarizes study design, sample, biomarkers, analyses, and key findings. |
| Introduction | | | |  |
| Background/rationale | 2 | Explain the scientific background and rationale for the investigation being reported | 6 - 7 | Introduction outlines evidence linking olfactory decline to dementia and discusses potential biological mechanisms |
| Objectives | 3 | State specific objectives, including any prespecified hypotheses | 6-7 | Aims include testing whether blood-based AD biomarkers predict longitudinal olfactory decline; hypothesis: biomarkers reflect underlying neuropathology linked to smell loss. |
| Methods | | | |  |
| Study design | 4 | Present key elements of study design early in the paper | 7 | Methods state in beginning that this is a longitudinal cohort study using SNAC-K data. |
| Setting | 5 | Describe the setting, locations, and relevant dates, including periods of recruitment, exposure, follow-up, and data collection | 7-10 | Study conducted in Kungsholmen, Stockholm; baseline 2001–2004 with follow-ups every 3 or 6 years depending on age group. |
| Participants | 6 | (*a*) *Cohort study*—Give the eligibility criteria, and the sources and methods of selection of participants. Describe methods of follow-up  *Case-control study*—Give the eligibility criteria, and the sources and methods of case ascertainment and control selection. Give the rationale for the choice of cases and controls  *Cross-sectional study*—Give the eligibility criteria, and the sources and methods of selection of participants | 8 | Inclusion: participants with baseline olfactory test and available biomarker data (n = 1144); follow-up smell tests at 6 and 12 years. |
|  |  | (*b*) *Cohort study*—For matched studies, give matching criteria and number of exposed and unexposed  *Case-control study*—For matched studies, give matching criteria and the number of controls per case | - | N/A – not a matched cohort design. |
| Variables | 7 | Clearly define all outcomes, exposures, predictors, potential confounders, and effect modifiers. Give diagnostic criteria, if applicable | 8-10 | Exposure: plasma biomarkers (p-tau217, p-tau181, NfL, etc.); Outcome: olfactory identification score; Covariates: age, sex, education in basic model. Age, sex, education, vocabulary, cardiovascular disease, chronic kidney disease, cerebrovascular disease, and anaemia in fully adjusted models. Effect modifiers: age group, sex, *APOE* ε4 carrier status, and smoking status |
| Data sources/ measurement | 8* | For each variable of interest, give sources of data and details of methods of assessment (measurement). Describe comparability of assessment methods if there is more than one group | 8-10 | Biomarkers measured using Simoa and MSD platforms; olfaction assessed via 16-item Sniffin' Sticks. |
| Bias | 9 | Describe any efforts to address potential sources of bias | 10 | Sensitivity analyses exclude a) those without any follow-up measure in olfaction and b) those with incident dementia; potential role of attrition or dementia addressed in discussion. |
| Study size | 10 | Explain how the study size was arrived at | 8 | Final sample size reflects available participants with valid olfaction and biomarker data; no a priori power calculation performed. |

Continued on next page

| Quantitative variables | 11 | Explain how quantitative variables were handled in the analyses. If applicable, describe which groupings were chosen and why | 9-10 | Biomarkers modeled continuously (log-transformed); olfactory score treated as continuous; quartiles of biomarkers used in secondary analyses. |
| --- | --- | --- | --- | --- |
| Statistical methods | 12 | (*a*) Describe all statistical methods, including those used to control for confounding | 9-10 | Mixed-effects linear regression; all multiadjusted models adjusted for age, sex, education, vocabulary, cardiovascular disease, chronic kidney disease, cerebrovascular disease, and anaemia. |
|  |  | (*b*) Describe any methods used to examine subgroups and interactions | 10 | Stratified analyses and interaction terms tested with potential effect modifiers (e.g., biomarker × time × age group); stratified models shown in supplementary material. Sensitivity analyses performed in participants without a) missing follow-up data and b) incident dementia during follow-up. |
|  |  | (*c*) Explain how missing data were addressed | 10 | Participants with missing olfaction or biomarker data excluded; missing covariate data <2%, handled by listwise deletion. |
|  |  | (*d*) *Cohort study*—If applicable, explain how loss to follow-up was addressed  *Case-control study*—If applicable, explain how matching of cases and controls was addressed  *Cross-sectional study*—If applicable, describe analytical methods taking account of sampling strategy | 10 | Dropout patterns described in Results; mixed models account for missing outcome data under maximum-likelihood assumption. |
|  |  | (*e*) Describe any sensitivity analyses | 10 | Sensitivity analyses exclude participants with a) no follow-up data; b) incident dementia; results discussed in Discussion. |
| Results | | | | |
| Participants | 13* | (a) Report numbers of individuals at each stage of study—eg numbers potentially eligible, examined for eligibility, confirmed eligible, included in the study, completing follow-up, and analysed | 8 | Sample flow described in Methods and Results; 1144 participants had olfactory and biomarker data at baseline. Follow-up olfaction data available for 822 (6 years) and 556 (12 years). |
|  |  | (b) Give reasons for non-participation at each stage | 8 | Loss to follow-up described as due to death, or dropout; see Methods and Results. |
|  |  | (c) Consider use of a flow diagram | - | No flow diagram included. Participant flow described in text. The sample is largely overlapping with that of a previous study on predictors of olfactory decline (Ekström et al., 2020), to which readers are referred for additional details on inclusion and follow-up. |
| Descriptive data | 14* | (a) Give characteristics of study participants (eg demographic, clinical, social) and information on exposures and potential confounders | Table 1 | Baseline characteristics presented for included participants. |
|  |  | (b) Indicate number of participants with missing data for each variable of interest | Table 1 | Percentage of missing data per variable noted in table footnotes. |
|  |  | (c) *Cohort study*—Summarise follow-up time (eg, average and total amount) | 8 | Olfactory follow-ups occurred at 3-year or 6-year intervals depending on age at baseline. Mean follow-up duration described in Methods. |
| Outcome data | 15* | *Cohort study*—Report numbers of outcome events or summary measures over time | 10, Table 2 | Olfactory scores over time reported across waves; change modeled using mixed-effects regressions. |
|  |  | *Case-control study—*Report numbers in each exposure category, or summary measures of exposure |  |  |
|  |  | *Cross-sectional study—*Report numbers of outcome events or summary measures |  |  |
| Main results | 16 | (*a*) Give unadjusted estimates and, if applicable, confounder-adjusted estimates and their precision (eg, 95% confidence interval). Make clear which confounders were adjusted for and why they were included | Table 2, 10–11 | Results section reports unadjusted estimates for olfactory decline. Tables report adjusted β coefficients with 95% CIs for each biomarker; covariates clearly specified. |
|  |  | (*b*) Report category boundaries when continuous variables were categorized | 12 | Quartiles of biomarkers in secondary analyses. |
|  |  | (*c*) If relevant, consider translating estimates of relative risk into absolute risk for a meaningful time period | - | Not applicable – outcome is olfactory score, not a risk estimate. |

Continued on next page

| Other analyses | 17 | Report other analyses done—eg analyses of subgroups and interactions, and sensitivity analyses | 10–11, Supplement | Interaction models (biomarker × time × effect modifier) and stratified analyses included; results reported in text and supplementary tables. |
| --- | --- | --- | --- | --- |
| Discussion | | | | |
| Key results | 18 | Summarise key results with reference to study objectives | 12 | Discussion section begins with summary of main findings, linking back to objectives. |
| Limitations | 19 | Discuss limitations of the study, taking into account sources of potential bias or imprecision. Discuss both direction and magnitude of any potential bias | 16 | Attrition addressed through sensitivity analyses restricted to participants with olfactory follow-up data, confirming longitudinal patterns. However, the sample may be healthier than the general population, potentially leading to conservative estimates. A key limitation is the unavailability of biomarkers for Lewy body pathology, despite its established role in olfactory dysfunction; no reliable blood-based assays for α-synuclein were available. |
| Interpretation | 20 | Give a cautious overall interpretation of results considering objectives, limitations, multiplicity of analyses, results from similar studies, and other relevant evidence | 16-17 | Cautious interpretation considering study limitations. |
| Generalisability | 21 | Discuss the generalisability (external validity) of the study results | 16 | Generalisability discussed in context of study sample being potentially healthier than general population. |
| Other information | |  | | |
| Funding | 22 | Give the source of funding and the role of the funders for the present study and, if applicable, for the original study on which the present article is based | 22 | Funders (Riksbankens Jubileumsfond, Demensfonden, Swedish Research Council) acknowledged; role in study design and reporting clarified as none. |

*Give information separately for cases and controls in case-control studies and, if applicable, for exposed and unexposed groups in cohort and cross-sectional studies.

**Note:** An Explanation and Elaboration article discusses each checklist item and gives methodological background and published examples of transparent reporting. The STROBE checklist is best used in conjunction with this article (freely available on the Web sites of PLoS Medicine at http://www.plosmedicine.org/, Annals of Internal Medicine at http://www.annals.org/, and Epidemiology at http://www.epidem.com/). Information on the STROBE Initiative is available at www.strobe-statement.org.
