# Supplementary material for: Blood-based biomarkers of Alzheimer’s disease and olfactory decline over 15 years in older adults
Source: GeroScience. 2025 Dec 17;48(5):7245–60. doi: 10.1007/s11357-025-02038-1 (PMC13601474; doi:10.1007/s11357-025-02038-1)
Supplement: Supplementary file 2 [file 11357_2025_2038_MOESM2_ESM.docx]

| **Supplementary Table 1.** Baseline Characteristics by Follow-up Status and by Dementia Status during Follow-up. | | | | | |
| --- | --- | --- | --- | --- | --- |
| **Characteristic** | **Total**  **(n = 1868)** | **No Follow-up**  **(n = 467)** | **Follow-up**  **(n = 1,401)** | **Dementia at follow-up**  **(n = 312)** | **No Dementia at follow-up**  **(n = 1,556)** |
| Odor Identification, M (SD) | 11.81 (2.93) | 11.19 (3.15) | 12.02 (2.83) | 10.07 (3.33) | 12.16 (2.72) |
| Range | 1 to 16 | 1 to 16 | 1 – 16 | 1 to 16 | 1 to 16 |
| Age, M (SD) | 71.29 (9.86) | 74.37 (10.70) | 70.27 (9.34) | 78.25 (7.77) | 69.90 (9.64) |
| Range | 58.44 to 100.9 | 60.13 to 100.92 | 58.44 to 96.69 | 60.2 to 96.7 | 58.44 to 100.92 |
| Education (years), M (SD) | 12.39 (4.24) | 11.36 (4.09) | 12.73 (4.23) | 11.27 (3.92) | 12.61 (4.27) |
| Range | 3 to 31.5 | 3 – 31.5 | 3 – 27.5 | 3 to 24 | 3 to 31.5 |
| MMSE, M (SD) | 28.95 (1.28) | 28.49 (1.61) | 29.10 (1.10) | 28.40 (1.48) | 29.06 (1.20) |
| Range | 24–30 | 24 – 30 | 24 – 30 | 24 to 30 | 24 to 30 |
| SRB, M (SD) | 23.06 (4.90) | 21.59 (5.66) | 23.55 (4.52) | 21.60 (5.16) | 23.35 (4.79) |
| Range | 1 to 30 | 1 – 30 | 3 – 30 | 3 to 30 | 1 to 30 |
| Sex, No. (%) - Men | 746 (39.9%) | 201 (43.0%) | 545 (38.9%) | 104 (33.3%) | 642 (41.3%) |
| Sex, No. (%) - Women | 1122 (60.1%) | 266 (57.0%) | 856 (61.1%) | 208 (66.7%) | 914 (58.7%) |
| Smoking, Current (%) | 289 (15.6%) | 96 (20.7%) | 193 (13.9%) | 40 (12.8%) | 249 (16.1%) |
| Smoking, Ever (%) | 746 (40.2%) | 176 (37.9%) | 570 (40.9%) | 117 (37.5%) | 629 (40.7%) |
| Smoking, Never (%) | 823 (44.3%) | 193 (41.5%) | 630 (45.2%) | 155 (49.7%) | 668 (43.2%) |
| APOE ε4 Status, No. (%) - No E4 | 1279 (70.2%) | 294 (68.5%) | 985 (70.8%) | 184 (59.9%) | 1,095 (72.3%) |
| APOE ε4 Status, No. (%) - E4 | 542 (29.8%) | 135 (31.5%) | 407 (29.2%) | 123 (40.1%) | 419 (27.7%) |
| Kidney Disease, No. (%) - Absent | 1323 (70.8%) | 293 (62.7%) | 1030 (73.5%) | 177 (56.7%) | 1,146 (73.7%) |
| Kidney Disease, No. (%) - Present | 545 (29.2%) | 174 (37.3%) | 371 (26.5%) | 135 (43.3%) | 410 (26.4%) |
| Cerebrovascular Disease, No. (%) - Absent | 1777 (95.1%) | 434 (92.9%) | 1343 (95.9%) | 283 (90.7%) | 1,494 (96.0%) |
| Cerebrovascular Disease, No. (%) - Present | 91 (4.9%) | 33 (7.1%) | 58 (4.1%) | 29 (9.3%) | 62 (4.0%) |
| Anemia, No. (%) - Absent | 1703 (91.2%) | 395 (84.6%) | 1308 (93.4%) | 271 (86.9%) | 1,432 (92.0%) |
| Anemia, No. (%) - Present | 165 (8.8%) | 72 (15.4%) | 93 (6.6%) | 41 (13.1%) | 124 (8.0%) |
| Obesity, No. (%) - Absent | 1610 (86.2%) | 404 (86.5%) | 1206 (86.1%) | 279 (89.4%) | 1,331 (85.5%) |
| Obesity, No. (%) - Present | 258 (13.8%) | 63 (13.5%) | 195 (13.9%) | 33 (10.6%) | 225 (14.5%) |
| Cardiovascular Disease, No. (%) - Score 0 | 1508 (80.7%) | 338 (72.4%) | 1170 (83.5%) | 218 (69.9%) | 1,290 (82.9%) |
| Cardiovascular Disease, No. (%) - Score 1 | 257 (13.8%) | 85 (18.2%) | 172 (12.3%) | 67 (21.5%) | 190 (12.2%) |
| Cardiovascular Disease, No. (%) - Score 2 | 83 (4.4%) | 34 (7.3%) | 49 (3.5%) | 21 (6.7%) | 62 (4.0%) |
| Cardiovascular Disease, No. (%) - Score 3 | 20 (1.1%) | 10 (2.1%) | 10 (0.7%) | 6 (1.9%) | 14 (0.9%) |

*Note.* Values are presented as mean (SD) or number (%). APOE ε4 status missing for 47 participants; smoking status missing for 10 participants.

| **Supplementary 2.** Results of Fully Adjusted Linear Models^a^ Estimating Odor Identification Scores as a Function of Biomarker Quartiles for Subsamples with Available Follow-up Data and without Dementia during Follow-up. | | | | | |
| --- | --- | --- | --- | --- | --- |
|  |  | **Subsample with Follow-up Data (n = 1394)** | | **Subsample without Dementia during Follow-up (n = 1546)** | |
|  |  | Baseline | Predictor × Time | Baseline | Predictor × Time |
|  |  | β (95% CI) | β (95% CI) | β (95% CI) | β (95% CI) |
| **Aβ42/Aβ40** | Q3 | 0.22 (-0.13, 0.57) | -0.01 (-0.05, 0.04) | 0.07 (-0.25, 0.38) | 0.01 (-0.04, 0.06) |
|  | Q2 | 0.12 (-0.24, 0.48) | -0.08 (-0.12, -0.03)** | 0.06 (-0.27, 0.40) | -0.06 (-0.11, -0.01)* |
|  | Q1 | 0.26 (-0.11, 0.63) | -0.09 (-0.14, -0.04)*** | -0.05 (-0.40, 0.29) | -0.05 (-0.10, 0.00) |
| **P-tau217** | Q1 | -0.16 (-0.51, 0.19) | -0.04 (-0.08, 0.01) | -0.15 (-0.47, 0.17) | -0.02 (-0.06, 0.03) |
|  | Q2 | -0.14 (-0.49, 0.22) | -0.10 (-0.15, -0.06)*** | -0.17 (-0.50, 0.16) | -0.07 (-0.11, -0.02)** |
|  | Q3 | -0.28 (-0.70, 0.13) | -0.20 (-0.25, -0.14)*** | -0.08 (-0.47, 0.31) | -0.15 (-0.21, -0.08)*** |
| **P-tau181** | Q1 | -0.10 (-0.44, 0.23) | -0.02 (-0.07, 0.02) | -0.12 (-0.43, 0.19) | 0.00 (-0.05, 0.04) |
|  | Q2 | -0.19 (-0.55, 0.16) | -0.08 (-0.13, -0.04)*** | -0.18 (-0.51, 0.15) | -0.05 (-0.10, -0.00)* |
|  | Q3 | -0.07 (-0.50, 0.36) | -0.18 (-0.24, -0.12)*** | 0.05 (-0.35, 0.45) | -0.17 (-0.24, -0.10)*** |
| **Total tau** | Q1 | 0.11 (-0.23, 0.46) | -0.01 (-0.06, 0.03) | -0.01 (-0.33, 0.31) | 0.00 (-0.05, 0.05) |
|  | Q2 | 0.11 (-0.24, 0.47) | -0.04 (-0.09, 0.01) | 0.05 (-0.28, 0.38) | -0.02 (-0.08, 0.03) |
|  | Q3 | -0.33 (-0.71, 0.05) | 0.01 (-0.04, 0.06) | -0.19 (-0.54, 0.15) | 0.02 (-0.04, 0.07) |
| **NfL** | Q1 | 0.13 (-0.20, 0.47) | -0.09 (-0.13, -0.04)*** | -0.14 (-0.44, 0.16) | -0.07 (-0.11, -0.03)** |
|  | Q2 | -0.06 (-0.46, 0.35) | -0.14 (-0.19, -0.09)*** | -0.03 (-0.40, 0.33) | -0.10 (-0.16, -0.05)*** |
|  | Q3 | -0.14 (-0.66, 0.38) | -0.24 (-0.30, -0.17)*** | -0.39 (-0.87, 0.09) | -0.20 (-0.28, -0.13)*** |
| **GFAP** | Q1 | -0.08 (-0.42, 0.25) | -0.02 (-0.06, 0.03) | -0.17 (-0.48, 0.14) | 0.00 (-0.04, 0.04) |
|  | Q2 | 0.09 (-0.28, 0.47) | -0.07 (-0.11, -0.02)** | -0.05 (-0.40, 0.29) | -0.06 (-0.11, -0.01)* |
|  | Q3 | -0.13 (-0.58, 0.32) | -0.18 (-0.25, -0.12)*** | -0.13 (-0.55, 0.30) | -0.12 (-0.20, -0.05)** |

*Note.* ****p* < 0.001, ***p* < 0.01, **p* < 0.05; CI = confidence interval. ^a^Adjusted for age, sex, years of education, vocabulary (SRB:1), chronic kidney disease, anaemia, cerebrovascular disease, and cardiovascular disease.

| **Supplementary Table 3.** Results of Basic and Multiadjusted Linear Models Estimating Odor Identification Scores as a Function of Continuous Biomarkers for the Total Sample. | | | | |
| --- | --- | --- | --- | --- |
|  | **Basic Adjusted Model^a^** | | **Fully Adjusted Model^b^** | |
|  | Baseline | Predictor × Time | Baseline | Predictor × Time |
|  | β (95% CI) | β (95% CI) | β (95% CI) | β (95% CI) |
| **Aβ42/Aβ40** | 0.02 (-0.09, 0.142) | 0.03 (0.01, 0.05)* | 0.02 (-0.10, 0.13) | 0.03 (0.01,0.05)* |
| **P-tau217** | -0.24 (-0.37, -0.12)*** | -0.17 (-0.21, -0.13)*** | -0.22 (-0.34, -0.10)** | -0.17 (-0.21, -0.13)*** |
| **P-tau181** | -0.20 (-0.35, -0.04)* | -0.08 (-0.12,- 0.05)*** | -0.16 (-0.32, -0.00)* | -0.08 (-0.12, -0.05)*** |
| **Total tau** | -0.13 (-0.24, -0.01)* | 0.00 (-0.02, 0.02) | -0.12 (-0.23 ,0.00) | 0.00 (-0.02, 0.02) |
| **NfL** | -0.05 (-0.20, 0.11) | -0.05 (-0.08, -0.02)*** | 0.01 (-0.15, 0.16) | -0.05 (-0.08, -0.03)*** |
| **GFAP** | 0.06 (-0.06, 0.19) | -0.02 (-0.04, -0.00)* | 0.06 (-0.07, 0.19) | -0.02 (-0.04, -0.00)* |

*Note.* ****p* < 0.001, ***p* < 0.01, **p* < 0.05; CI = confidence interval. ^a^Adjusted for age, sex, and years of education. *N* = 1867. ^b^Adjusted for age, sex, years of education, vocabulary (SRB:1), chronic kidney disease, anaemia, cerebrovascular disease, and cardiovascular disease. *N* = 1856.
